# Editorial: Towards the embedding of artificial intelligence into synthetic organisms: engineering intelligence in microorganisms

**DOI:** 10.3389/fgene.2025.1562092

**Published:** 2025-02-05

**Authors:** Martín Eduardo Gutiérrez, Rafael Lahoz-Beltrá, Alberto J. Donayre-Torres

**Affiliations:** ^1^ School of Informatics and Telecommunications, Faculty of Engineering and Sciences, Diego Portales University, Santiago, Chile; ^2^ Department of Biodiversity, Ecology and Evolution (Biomathematics), Faculty of Biological Sciences, Complutense University of Madrid, Madrid, Spain; ^3^ Bioengineering and Chemical Engineering Department, Universidad de Ingeniería y Tecnología, Lima, Peru

**Keywords:** synthetic and systems biology, artificial intelligence, in-cell, algorithms, gene network

The main goal of this Research Topic was to gather and foster work that describes the design and/or implementation of AI within microorganisms, enabling them to use data in their environment to make autonomous decisions on a basic level. The technology for implementing such mechanisms lies within synthetic biology. Implementations of the mentioned AI methods should also be understood as “biological versions” of the computational algorithms, implying that living components and processes are made part of those versions of the algorithms, harnessing the intrinsic operation and processing power of microorganisms.

Four works were published within this Research Topic, in chronological order:

- An opinion article “*Recurrent neural networks in synthetic cells: a route to autonomous molecular agents?*” by Braccini et al. The article first reviews previous definitions and implementations of chemical neural networks (CNNs). Such networks operate differently than computational artificial neural networks, in the sense that they are only alike in structure, but mix computation and computed information. Also, these networks exist and work within cells. From here, the authors propose recurrent neural networks as a direction to endow cells with a certain degree of autonomy. The authors also comment on the constraints of implementing recurrence in CNNs. Finally, they conclude that autonomy is a requisite for advancing chemical based AI, but also recognize the difficulty in its implementation. In sum, this manuscript highlights important concepts and existing gaps to address advancement towards full in-cell AI.
- A research article “*Closing the loop on morphogenesis: a mathematical model of morphogenesis by closed-loop reaction-diffusion*,” by Grodstein et al. This article reports on the design and implementation of simulations that endow bacteria with the capacity to sense their environment and signals to artificially replicate reaction-diffusion systems with a given number of peaks, based on closed negative feedback loops. It is implemented within a genetic regulatory network. This artificially constructed solution seeks to control morphogenesis within cell colonies and demonstrates an emergence of global patterning and coordination through signals and environment. Thus, this article showcases an example of how intelligent behavior (such as the cell counting the number of reaction-diffusion peaks in a given direction to respond with its own action) may be inserted into a cell.
- A review article “*Intelligent computation in cancer gene therapy*” by Samuel and Daniel. This article establishes a base level required for intelligent computation (oriented towards cancer gene therapy) in biological computation: more specifically, highlighting digital computation (binary classification, use of AND gates) as the standard in cancer gene therapy. Nonetheless, neuromorphic computation is presented by the authors as the next step to overcome limitations associated to digital based solutions, identifying analog computation as the major improvement introduced by this form of computation. This review article identifies and links well-known AI concepts and algorithms to current solutions in cancer gene therapy. The reviewed solutions focus on microorganisms as hosts and executing agents of the algorithms.
- A research article “*Genomic analysis and mechanisms exploration of a stress tolerance and high-yield pullulan producing strain*” by Yang et al. This article reports on molecular mechanisms for optimizing the production of pullulan in *Aureobasidium melanogenum* TN3-1 strain. The text documents the whole process in which the genome was characterized and its evolution (as a form of evolutionary algorithm) was studied to understand the optimization mechanism for the pullulan yield. The manuscript narrates evolutionary algorithms within cells, but from a purely biological and genomic perspective, adding an interesting view on in-cell AI.

We hope that the Research Topic fulfilled its goal in attracting interest and bringing to the attention of researchers from all fields related to AI, synthetic biology, and genomics that in-cell AI implementation is a difficult endeavor, but entails great opportunities in its applications. The editors of this topic hope that you enjoy it, and find in it a useful and novel resource related to in-cell AI, and that it motivates more researchers to undertake this difficult, but also rewarding research path.

